# Methods for Simultaneous Robot-World-Hand–Eye Calibration: A Comparative Study

**DOI:** 10.3390/s19122837

**Published:** 2019-06-25

**Authors:** Ihtisham Ali, Olli Suominen, Atanas Gotchev, Emilio Ruiz Morales

**Affiliations:** 1 Faculty of Information Technology and Communication, Tampere University, 33720 Tampere, Finland; olli.j.suominen@tuni.fi (O.S.); atanas.gotchev@tuni.fi (A.G.); 2 Fusion for Energy (F4E), ITER Delivery Department, Remote Handling Project Team, 08019 Barcelona, Spain; Emilio.Ruiz@f4e.europa.eu

**Keywords:** robot-world-hand–eye calibration, hand–eye calibration, optimization

## Abstract

In this paper, we propose two novel methods for robot-world-hand–eye calibration and provide a comparative analysis against six state-of-the-art methods. We examine the calibration problem from two alternative geometrical interpretations, called ‘hand–eye’ and ‘robot-world-hand–eye’, respectively. The study analyses the effects of specifying the objective function as pose error or reprojection error minimization problem. We provide three real and three simulated datasets with rendered images as part of the study. In addition, we propose a robotic arm error modeling approach to be used along with the simulated datasets for generating a realistic response. The tests on simulated data are performed in both ideal cases and with pseudo-realistic robotic arm pose and visual noise. Our methods show significant improvement and robustness on many metrics in various scenarios compared to state-of-the-art methods.

## 1. Introduction

Hand–eye calibration is an essential component of vision-based robot control also known as visual servoing. Visual servoing effectively uses visual information from the camera as feedback to plan and control action and motion for various applications such as robotic grasping [1] and medical procedures [2]. All such applications require accurate hand–eye calibration primarily to complement the accurate robotic arm pose with the sensor-based measurement of the observed environment into a more complete set of information.

Hand–eye calibration requires accurate estimation of the homogenous transformation between the robot hand/end-effector and the optical frame of the camera affixed to the end effector. The problem can be formulated as AX=XB, where A and B are the robotic arm and camera poses between two successive time frames, respectively, and X is the unknown transform between the robot hand (end effector) and the camera [3,4]. 

Alternatively, the estimation of a homogeneous transformation from the robot base to the calibration pattern/world coordinate system can be obtained as a byproduct of the problem solution widely known as robot-world-hand–eye (RWHE) calibration, formulated as  AX=ZB. In this formulation, we define X as the transformation from robot base to world/pattern coordinate and Z is the transformation from the tool center point (TCP) to the camera frame. These two notations might be opposite in some other studies. The transformations A and B no longer represent the relative motion poses between different time instants. Instead, they now represent the transformation from TCP to the robot base frame, and the transformation from the camera to the world frame. 

A considerable number of studies have been carried out to solve the problem of hand–eye calibration. While the core problem has been well addressed, the need for improved accuracy and robustness has increased with time as the hand–eye calibration problem expands to finds its uses in various fields of science.

The earliest approach presented for hand–eye calibration estimated the rotational and translational parts individually. Due to the nature of the approach, the solution is known as *separable* solution. Shiu and Ahmed [4] presented a closed-form approach to finding the solution for the problem formulation AX=XB by separately estimating the rotation and translation from robot wrist to the camera in that order. The drawback of the approach presented was that the linear system doubles at each new entry of the image frame. Tsai [3] approached the problem from the same perspective, however, they improved the efficiency of the method by keeping the number of unknowns fixed irrespective of the number of images and robot poses. Moreover, the derivation is both simpler and computationally efficient compared to [4]. Zhuang [5] adopted the quaternion representation for solving the rotation transformation from hand to eye and robot base to the world. The translation components are then computed using linear least squares. Liang et al. [6] proposed a closed-form solution by linearly decomposing the relative poses. The implementation is relatively simple; however, the approach is not robust to noise in the measurements and suffers intensely in terms of accuracy. Hirsh et al. [7] proposed a separable approach that solves for X and Z alternatingly in an iterative process. The approach makes an assumption that one of the unknown is pseudo-known for that time being and estimates the best possible values for the other unknown by distributing the error. In the first case, it assumes that Z is known by the system and estimates X by averaging over the equation $X=ZB_{n}A^{-1}$ for all n poses of B. Similarly, an estimation for Z is obtained by using the previously obtained X. This process continues until the system reaches the condition to terminate the iterative estimation. In a recent study, Shah [8] proposed a separable approach that forms its bases on the methods presented by Li et al. [9]. Shah suggests using the Kronecker product to solve the hand–eye calibration problem. The method first computes the rotational matrices for the unknown X, followed by computing the translation vectors. Kronecker product is an effective approach to estimate the optimal transformation in this problem. However, the resulting rotational matrices might not follow orthogonality. To compensate for this issue, the best approximations for orthonormal rotational matrices are obtained using Singular Value Decomposition (SVD). The primary difference between the work of [8] and [9] is that Li et al. do not update the positions that were only optimal for the rotational transformation before the orthonormal approximation. This augments to any errors that might already be present in the solution. In contrast, Shah [8] explicitly re-computes the translations based on the new orthonormal approximations of the rotations $R_{X}$ and $R_{Z}$. Earlier studies have shown that separable approaches have a core limitation, which results in slightly high position errors. Since the orientations and translation are computed independently and in the mentioned order, the errors from orientations step propagate to the position estimation step. Typically, separate solution based approaches have good orientation accuracy; however, the position accuracy is often compromised.

The second class of solutions is based on *simultaneous* computation of the orientation and position. Chen [10] argued that rotation and translation are interdependent quantities and, therefore, should not be estimated separately. He proposed a simultaneous approach to the hand–eye problem based on screw theory where both the rotation and translation components are computed altogether. In his work, Chen estimates a rigid transformation to align the camera screw axis to the robot screw axis. Dornaika and Horaud [11], proposed a nonlinear least square based approach to solve the hand–eye calibration problem. The optimization approach solved for an abundant number of parameters that represent rotations in the form of matrices. The cost function constrained the optimization to solve for orthonormal rotation matrices. It was observed that the nonlinear iterative approach yielded better results to linear and closed form solution in term of accuracy. Henceforth, many studies have opted for nonlinear cost minimization approach since they are more tolerant to nonlinearities present in measurements in the form of noise and errors. Shi et al. [12] proposed to replace the rotation matrices with quaternion representation to facilitate the iterative optimization approach towards a solution. In [13], Wei et al. contributed an approach for an online hand–eye calibration approach that estimate the transformations through active motion. The method discards degenerative cases where no or little rotation cases induce high errors into the system. Strobel and Hirzinger [14], proposed an adaptive technique for hand–eye calibration using nonlinear optimization. The approach adjusts weights that are assigned to the rotation and translation errors during the cost minimization step. In [15], Fassi and Legnai construed a geometrical interpretation of the hand–eye calibration problem for the formulation AX=XB. They argued that the general formulation can lead to an infinite solution and therefore a constrained multi-equation based system is always suitable to optimize. Some cases that result in singularity were also discussed. Zhao [16] presents a convex cost function by employing the Kronecker product in both rotational matrix and quaternion form. The study argues that a global solution can be obtained using linear optimization without specifying any initial points. This serves as an advantage over using L2 based optimization. Heller et al. [17] proposed a solution to the hand–eye calibration problem using the branch-and-bound (BnB) method introduced in [18]. The authors minimize the cost function under the epipolar constraints and claim to yield a globally optimum solution with respect to $L_{\infty }-$norm. Tabb [19] tackled the problem of hand–eye calibration from the iterative optimization based approach and compared the performance of various objective functions. The study focused on AX=ZB formulation and solved for the orientation and translation both separately and simultaneously using the nonlinear optimizer. Moreover, a variety of rotation representations was adopted including Euler, rotation matrix and quaternion in order to study their effect on accuracy. The study explored the possibility of a robust and accurate solution by minimizing pose and reprojection errors using different costs. The authors used the nonlinear optimizer Ceres [20] to solve for a solution using the Levenberg-Marquardt algorithm.

In this study, we present a collection of iterative methods for the hand–eye calibration problem under both AX=XB and AX=ZB formulations. We adopt the iterative cost minimization based approach similar to Tabb [19]. However, the geometrical formulation is reverted to the generic form for better coherence. Moreover, we study the problem from AX=XB formulation, which is not present in [19]. The prospects of a new cost functions for the non-linear regression step are also studied. Each method is quantified from pose optimization and reprojection error minimization perspective. The main contributions of this study are as follows:

(1) We provide a comprehensive analysis and comparison of various cost functions for various problem formulations.
(2) We provide a dataset composed of three simulated sequences and three real data sequence, which we believe is handful for testing and validation by the research community. To the best of our knowledge, this is the first simulated data set for hand–eye calibration with synthetic images that are available for public use. Moreover, the real data sequences include chess and ChArUco calibration board of varying sizes. The datasets are available from [21].
(3) We provide extensive testing and validation results on a simulated dataset with realistic robot (position and orientation) and camera noise to allow comparisons between the estimated and true solutions more accurately.
(4) We provide an open-source code of the implementation of this study along with the surveyed approaches to support reproducible research. The code is available from [21].

The article is organized as follows: In Section 2, we present in detail the problem formulations for robot-world-hand–eye calibration. In Section 3, we discuss the development of real and synthetic dataset for evaluation purpose. Section 4 presents the error metrics used to quantify the performance of the calibration methods. Section 5 summarizes the experimental results using both synthetic and real datasets against the aforementioned error metrics. Finally, Section 6 concludes the article.

## 2. Methods

**Figure 1:**
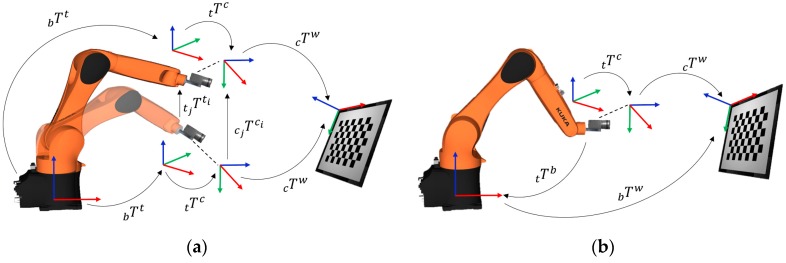
Formulations relating geometrical transformation for calibration; (**a**) hand–eye calibration; (**b**) robot-world-hand–eye Calibration.

For the needs of our study, we introduce notations, as illustrated in Figure 1. Throughout this article, we will represent homogenous transformations by T supported with various sub-indexes. The sub-indexes *b*, *t*, *c* and *w* indicate the coordinate frames associated with robot base, robot tool, camera and the calibration pattern, respectively. The sub-indexes *i* and *j* are associated with time instants of the state of the system. For the first general formulation AX=XB illustrated in Figure 1a, Tbbi is the equivalent to $A_{i}$ and denotes the homogenous transformations from robot base to the tool center point (TCP)/end-effector. Tciw is the equivalent of $B_{i}$ and denotes the homogenous transformation from camera to the world/calibration pattern. The formulation uses the relative transformation A (Ttjti). and B (Tcjci) from their respective previous pose to another pose. The unknown X or Ttc is the required homogenous transformation from the end effector to the camera.

The second general formulation, AX=ZB is illustrated in Figure 1b. The formulation uses absolute transformation A(Ttb) and B(Tcw) from their respective coordinate frames. The unknown X(Tbw) and Z(Ttc) are the homogenous transformations from robot base to the world frame and the end effector to the camera frame, respectively. The hand–eye transformation is referred to as **Z** in this formulation for coherence in literature, since many studies opt for such notation.

In this section, we focus on various cost functions for the two general problem formulations with the aim to analyze their performance under real situations. For both cases, we consider solving the problem by minimizing pose error and reprojection error. Some studies including [19] propose to optimize the camera’s intrinsic parameters using the nonlinear solver to yield better results. However, Koide and Menegatti [22] argue that the approach involving camera intrinsic optimization overfits the model on the data for the reprojection error; consequently, the results are poor for other error metrics including reconstruction accuracy. Following the insight from [22], we solve for the transformation by minimizing the reprojection error.

The main information required for hand–eye calibration are the Tool Centre Point (TCP)/end effector poses and the camera poses. The TCP pose of the robotic arm is directly provided by the software of the robotic arm against the base of the arm. The pose is typically quite accurate due to the high accuracy of the encoders in the robotic arm that provide feedback for the angles of the joints. In general, for many robotic arms, the precision for the end effector’s position is around 0.1–0.2 mm. On the other hand, the camera pose against the world frame can be obtained through various methods. The common approach is to use a calibration pattern for simultaneously calculating the calibration parameters of the camera and the pose of the camera against the pattern or in this case world frame. Many researchers favor this approach since the calibration pattern is easy to acquire and its use yields good results. In contrast, some studies [23,24] prefer Structure from Motion (SFM) to acquire the relative camera transformation when the camera is moved from one point to another. The approach is independent of the calibration pattern and can acquire the correspondences from the feature-rich environment. However, SFM based camera calibration and camera pose computation are prone to errors. The approach inherits additional errors into the hand–eye calibration process and reduces the overall accuracy of the system. To compensate for these errors, the process must include additional steps to mitigate the effects. The added efforts deviate the focus from the core target, which is accurate hand–eye calibration. In this study, we utilize industrial-grade calibration boards in order to estimate the camera intrinsic parameters and camera extrinsics for the robot-world-hand–eye calibration problem. The camera calibration approach used in this study is based on the widely adopted method by Zhang [25].

### 2.1. Hand–Eye Formulation

This mathematical problem formulation involves estimating one unknown with the help of two known homogenous transformations in a single equation. Let Tbti be the homogenous transformation from the base of the robot to the robot TCP. The homogenous transformation relating the camera coordinate frame to the world coordinate frame affixed to the calibration patters is Tciw. The unknown homogenous transformation from the tool to the camera coordinate frame to be estimated is represented by Ttc. Then from Figure 1a, we can form the following relationship

(1) Tbt2−1 Tbt1  Ttc=Ttc  Tc2w Tc1w−1 ← (Tt1c1=Tt2c2)

(2) Tt2b Tbt1  Ttc=Ttc  Tc2w Twc1. 

Generalizing Equation (2) gives us Equation (3).

(3) Ttjti  Ttc=Ttc Tcjci

(4) [RtjtiTtjti01×31] [Rtcttc01×31] = [Rtcttc01×31] [Rcjcitcjci01×31]

Equation (4) represents the direct geometrical relationship between various coordinate frames involved in the system. In order to attain a solution and achieve dependable results it is required that the data is recorded for at least 3 positions with non-parallel movements of the rotational axis [14]. We can directly minimize the relationship in Equation (4) to estimate the unknown parameters presented in Equation (5). In the experimentation section, we refer to the cost functions in Equations (5) and (6) as Xc1 and Xc2, respectively.

(5) {q(t,c),ttc} = argminq(t,c) , ttc∑i=1,j=i+1n−1||n¯( Ttjti  [q(t,c),ttc]HT−[q(t,c),ttc]HTT cjci )||22

In light of recommendation of [19], we can also re-arrange Equation (5) in the following manner.

(6) {q(t,c),ttc} = argminq(t,c) , ttc∑i=1,j=i+1n−1||n¯( Ttjti −[q(t,c),ttc]HTT cjci  [q˜(t,c),t˜tc]HT)||22

Here, the symbol ${\left[\right]}_{HT}$ denotes the conversion of the parameters to homogenous transformation representation. The solver optimizes the parameters in quaternion representation$\;\;q_{\left(t,c\right)}$ of the rotational matrix Rtc and translation ttc. The operation $\bar{n}$ denotes the aggregation of the 4 × 4-error matrix into a scalar value by summation of normalized values of quaternion angles and normalized translation vector. The terms ${\tilde{q}}_{\left(t,c\right)}$ and t˜tc are the quaternion and translation vector obtained from the inverse of Ttc. The objective functions minimize the L2-norm of the residual scaler values. The solutions in Equations (5) and (6) belong to the simultaneous solution category of hand–eye calibration because the rotation and translation are solved at the same time. We use the Levenberg –Marquardt algorithm to search for a minimum in the search space. The objective function successfully converges to a solution without any initial estimates for the $q_{\left(t,c\right)}$ and ttc. We have observed that the cost function in Equation (6) enjoys a slight improvement in some cases over Equation (5), which will be discussed in the experimental results and discussion section.

The second approach to seek a solution is based on reprojection-based methods. Reprojection error minimization has shown promising results for pose estimation in various problem cases [26,27]. Tabb [19] examined the reprojection-based method for the AX=ZB formulation. We generalize this approach for the case of the AX=XB formulation. Let W be the 3D points in the world frame and $P^{c}\;$be the same points in the camera frame. In the case of the chessboard pattern, these points are the corners of the chessboard. The following relationship represents the objective function for minimizing the reprojection error of the 3D points from pose *i* to pose *j*. The cost function in Equation (7) is referred to as RX here onwards.

(7) {q(t,c),ttc} =argminq(t,c) , ttc∑i=1,j=i+1n−1 ||P¯j   −Π(   K,   [q˜(t,c),t˜tc]HT T tjti [q(t,c),ttc]HT ,  Pic)||22

In the equation, Π represents the operation that projects the 3D points from world space to image space using the camera intrinsic K and the camera extrinsic obtained using the homogenous transformations given in Equation (7), while ${\bar{P}}_{j}$ are the observed 2D points in the j-th image. 

It is important to note that the reprojection error minimization based approach is not invariant to the choice of initial estimates for the solver. However, if a good initial estimate is provided, the nonlinear optimization of reprojection error can provide a more accurate solution with a fine resolution.

### 2.2. Robot-World-Hand–Eye Formulation

This mathematical formulation involves the estimation of an additional homogenous transformation that is between the robot base frame and world frame. Therefore, we have two known and two unknown homogenous transformations. Let Ttb. be the homogenous transformation from robot TCP to the base of the robot. The homogenous transformation relating the camera coordinate frame to the world coordinate is Tcw.The additional unknown homogenous transformation from the robot base frame to the world frame is Tbw. Then from Figure 1b, we can form a straightforward geometrical relationship as:

(8) TtbTbw=Ttc Tcw

(9) [Rtbttb01×31] [Rbwtbw01×31] =[Rtcttc01×31] [Rcwtcw01×31]

Similar to the previous cases, we can directly use the relationship in aforementioned equations to obtain Ttc and Tbw using nonlinear minimization of their respective costs

(10) {q(t,c),ttc,q(b,w),tbw} =argminq(t,c) , ttc, q(b,w),tbw∑i=1n||n¯( Ttib  [q(b,w),tbw]HT−[q(t,c),ttc]HTT ciw )||22

We can observe from Equation (10), that we are attempting to solve for two unknown homogenous transformations. The adopted parametrization involves optimizing over 14 parameters, where the two quaternions and translation vectors contribute to 8 and 6 parameters, respectively. While the robot-world-hand–eye calibration involves more unknowns for estimation, nonetheless, it constrains the geometry with more anchor points and helps to converge closer to the global minimum. With the advent of modern nonlinear solvers, the problem of optimizing for a large number of unknowns has become simpler and more efficient. As before, the objective function in Equation (10) can be re-arranged in the form of Equation (11). The cost functions in Equations (10) and (11) are referred to as Zc1 and Zc2, respectively, in Tabb [19]

(11) {q(t,c),ttc,q(b,w),tbw} =argminq(t,c) , ttc, q(b,w),tbw∑i=1n||n¯( Ttib − [q(t,c),ttc]HT T ciw [q˜(b,w),t˜bw]HT)||22

The objective function successfully converges to a solution for q(t,c),ttc,q(b,w) and tbw. However, the primary difference here is that the solver depends on initialization. In case of bad initial estimates, the optimization algorithm might not converge to a stable solution. However, the formulation presented is not a high dimensional optimization problem and therefore, a rough initial estimate is sufficient. The initial estimates can be acquired from any fast closed-form method such as Tsai [3] or Shah [8].

This formulation can also be viewed as reprojection error minimization problem. The following equation presents a cost function that minimizes the reprojection of the 3D world points W onto the image space in camera frame, where$\;{\bar{P}}_{i}$ are the observed 2D points in the *i*-th image. The cost functions in Equation (12) is referred to as rp1 in [19].

(12) {q(t,c),ttc,q(b,w),tbw} =argminq(t,c) , ttc, q(b,w),tbw∑i=1n ||P¯i−Π( K,  [q˜(t,c),t˜tc]HT Ttib [q(b,w),tbw]HT, W)||22

In contrast to the reprojection error cost function for problem formulation =XB, this formulation from [19] has the added advantage that it is not explicitly affected by the errors in pose estimation caused by blurred images or low camera resolution. If the camera intrinsic parameters are accurate enough, then the extrinsic can be indirectly computed as a transformation through Ttc, Ttb and Tbw through the minimization of the objective function. On the contrary, the reprojection error cost function presented for problem formulation AX=XB is more robust to robot pose errors given good images.

A marginal improvement in the results can be observed in various cases by using log(cosh(x)) as the loss function. The relative improvement is discussed in detail in Section 5. log(cosh(x)) approximates $\frac{x^{2}}{2}$ for small value of x and abs(x)−log(2), for large values. This essentially means that log(cosh(x)) imitates the behavior of the mean squared error but is more robust to noise and outliers. Moreover, the function is twice differentiable everywhere and therefore does not deteriorate the convexity of the problem. The modified version is given as followed, where E(x) is the error in terms of difference between the observed points and the reprojected points. The cost function in Equation (13) is referred to as RZ hereafter.

(13) {q(t,c),ttc,q(b,w),tbw} =argminq(t,c) , ttc, q(b,w),tbw∑i=1n|| log(cosh( E(x)))||22

## 3. Performance Evaluation Using Datasets

In order to assess the performance of the robot-world-hand–eye calibration methods, we present multiple datasets to test the methods in laboratory and near field settings. These datasets contain data acquired using various combinations of camera, lens, calibration patterns and robot poses. A detailed description of datasets is provided in Table 1. The table also lists the length of each side of square of the calibration patterns, focal length of the lenses, and number of robot poses used to acquire images. The datasets include real data and simulated data with synthetic images. To the best of our knowledge, this study is the first to provide simulated robot-world-hand–eye calibration dataset with synthetic/rendered images as open source for public use. A more detailed explanation of the datasets is presented in the following subsections. 

### 3.1. Real Datasets

**Figure 2:**
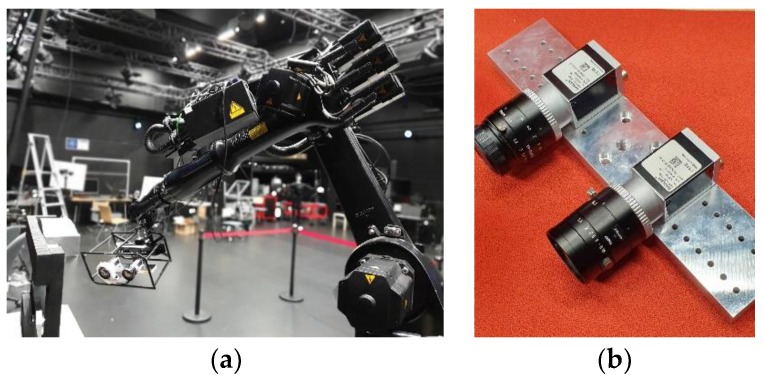
An example of the setup for acquiring the datasets; (**a**) robotic arm moving in the workspace; (**b**) cameras and Lenses for data acquisition.

**Figure 3:**
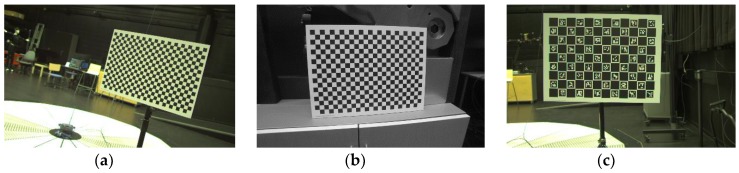
Example of captured images from the dataset 1 through 3; (**a**) checkerboard from dataset 1; (**b**) checkerboard from dataset 2; (**c**) ChArUco from dataset 3.

To acquire real data for this experiment, a KUKA KR16L6-2 serial 6-DOF robot arm was used with Basler acA1920-50gc camera using 12 mm and 16 mm lenses as shown in Figure 2. The primary aim in recording these datasets was to collect real data for various robot-world-hand–eye calibration tests. With this aim, the collection provides three real datasets with varying robot poses and calibration patterns as shown in Figure 3. In this study, we primarily use the chessboard pattern for accurate camera calibration and robot-world-hand–eye calibration. A minor yet significant difference between the datasets [28], used in [19], is that the robot hand/camera orientation changes are quite gentle. This is done to facilitate the OpenCV camera calibration implementation used in [19], therefore the aforementioned implementation is not invariant to significant orientation changes and as a result, it flips the origin of the calibration pattern. For our experiments, we utilized MATLAB’s implementation of [25], which can correctly detect the orientation of the pattern in any given pose. However, this neat trick requires that the calibration pattern is asymmetric in the number of rows and columns and that one of the sides has an even number of squares while the other side has odd. This requirement makes the datasets in [28], which have chessboard patterns with even number of rows and columns, unusable in our tests.

In addition, the calibration board used in the third dataset is a ChArUco pattern with square size of 60 mm, shown in Figure 3c. ChArUco tries to combine the benefit of both chessboard and ArUco markers and tends to facilitate the calibration process by fast, robust and accurate corner detection even in occluded or partial views [29]. The ChArUco dataset is only provided as open source material for future testing and has not been utilized in this study.

### 3.2. Simulated Dataset with Synthetic Images

**Figure 4:**
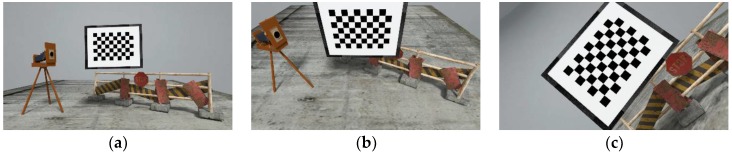
Example of rendered images for simulated datasets from the datasets 4 through 6; (**a**) excerpt from dataset 4; (**b**) excerpt from dataset 5; (**c**) excerpt from dataset 6.

The real data has the advantage of encapsulating all the uncertainties of a real system; however, in such cases we do not have any ground truth information. It is not possible to acquire the ground truth TCP-to-camera transformation, since the camera frame lies inside the camera. While various metric for relative errors and error distribution can be used, nonetheless, the absolute pose error is always missing to quantify accuracy. The main purpose of using simulated data is to quantify the accuracy of the estimated poses against ground truth pose for various robot-world-hand–eye calibration methods. We provide three simulated datasets as part of the dataset package excerpts of which are shown in Figure 4. Each dataset provides different number of poses and complexity through the orientation of the camera. The simulated data comprises of synthetic images generated in Blender [30], a 3D computer graphics software, of the specifications mentioned in Table 1. For simplification, we assume that the camera position is the same, as the robot TCP position. Then the homogenous transformation from hand-to-eye constitutes of rotation resulting from the orientation difference between the Blender world frame and Blender camera frame.

### 3.3. Pseudo-Real Noise Modeling

While simulated data carries the advantage of providing the ground truth information for various robot-world-hand–eye calibration, the limitation is that it lacks the uncertainties of the real world situations. These uncertainties could originate from either robot TCP pose errors or camera pose errors. Many studies [19,22,31] suggest testing the robustness of the methods by inducing one type of noise at a time into the system and evaluating its performance based on the response. Unfortunately, these uncertainties are mostly co-existent and co-dependent in real-world cases. In this study, we propose to model the uncertainties in terms of pose and pixel errors and induce a realistic amount of noise simultaneously into the simulated dataset for testing. The motivation behind inducing such type of noise is to carry the advantage of testing simulated data for accuracy and adjoining it with the robustness of testing on real data.

We aim at introducing a realistic amount of noise. The robot position repeatability is generally provided in the datasheets, which ranges from 0.1–0.3 mm for various robots. However, the orientation repeatability is absent since it cannot be measured for real robots at such a fine resolution. Here, we propose a reverse engineering approach to acquire a statistically valid amount of orientation noise. The position and orientation error of the TCP arises from the accumulated errors of the individual joints of the robotic arm due to robot flexibility and backlash. Using inverse kinematic we can find the joint angles for any position of TCP within its workspace.

**Figure 5:**
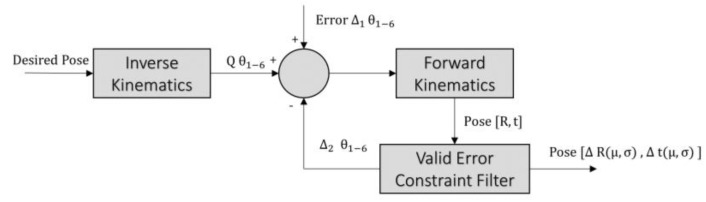
Flowchart of the orientation noise modelling approach.

Once the joint angles are available, we can introduce noise into the individual joints through trial and error until it produces the end-effector position error comparable to the realistic error. Through forward kinematics, we can then estimate the position and orientation of the end-effector under various arm configurations. Figure 5 shows the operation flow for computing the error range of the new orientations.

**Figure 6:**
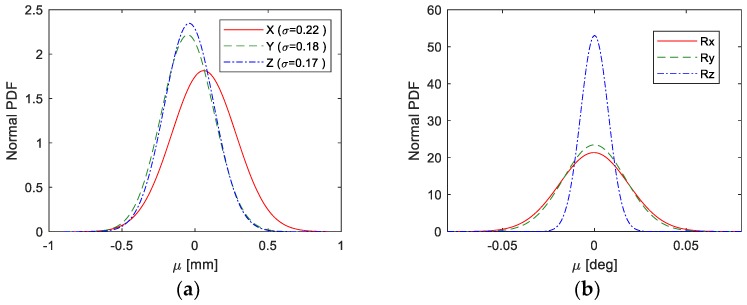
Probability distributions functions; (**a**) the measured position error from the robotic arm; (**b**) the modeled orientation error for the robotic arm.

For our test, we used the position error of the KUKA KR16L6-2 computed through highly accurate laser sensor. The mean of the errors in X, Y and Z axes were 0.06 mm, –0.05 mm and –0.04 mm, while the standard deviation of the errors were 0.22 mm, 0.18 mm and 0.17 mm. A normally distributed error for each axis is generated based on these values and introduced to the system to estimate the corresponding effects in the orientation of the TCP. The range of realistic valid error for the TCP position is shown in Figure 6a, while the output of the orientation error using the aforementioned framework is shown in Figure 6b.

## 4. Error Metrics

In order to compare the results of all the methods with other existing studies, we suggest to use mean rotation error (deg), mean translation error (mm), reprojection error (px), absolute rotation error (deg), and absolute translation error (mm). Each error metric has its own merits and demerits. We have avoided the use of reconstruction error since it involves further estimation of valid 3D points from the reprojected 2D points. This can be achieved by searching the space for such 3D points using nonlinear minimization, as before. However, it is not possible to segregate the error that arises from the pose estimation step and the reconstruction step, while using the error metric.

The first error is the mean rotation error derived from Equations (4) and (9) for AX=XB and AX=ZB formulation, respectively. Equation (16) gives the mean rotation error, which takes its input from Equations (14) and (15) for their respective formulation. Here, the angle represents the conversion from a rotation matrix to axis-angle for simpler user interpretation.

(14) ΔR=(Rtc Rcw )−1RtbRbw

(15) ΔR=(Rtc Rcjci )−1RtjtiRtc

(16) $$e_{rR}=\frac{1}{n}\overset{n}{\underset{i=1}{\sum }}||angle\left(\Delta R\right){||}_{2}^{2}$$

The second error metric focuses on computing the translation errors. As above, the translation errors emerge from the same Equations (4) and (9).

(17) ert=1n∑i=1,j=i+1n−1||(Rtjti ttc)+ ttjti−(Rtc tcjci)+ttc||22

(18) ert=1n∑i=1n||(Rtb tbw)+ ttb−(Rtc tcw)+ttc||22

The third metric to measure the quality of the results is the reprojection root mean squared error (rrmse). The rrmse is measured in pixels and is a good metric to measure the quality of the results in the absence of ground truth information. The rrmse provides an added advantage that it back-projects the 3D points from the calibration board onto the images by first transforming them through the robotic arm. In case, if the hand eye calibration is not correct, the reprojection errors will be large. The rrmse for both the formulations are given in Equations (19) and (20).

(19) errmse=1n−1∑i=1,j=i+1n−1||P¯j −Π( K,  [q˜(t,c),t˜tc]HT T tjti [q(t,c),ttc]HT ,  Pic)||22

(20) errmse=1n∑i=1n||P¯i −Π( K,  [q˜(t,c),t˜tc]HT Ttib [q(b,W),tbw]HT ,  W)||22

For the case of simulated data, we have accurate ground truth pose from the robot TCP to the camera. We can effectively utilize that information to acquire the absolute rotation error and absolute translation errors. The absolute rotation error can be obtained using Equation (21), while the absolute translation error is given using Equation (22). Here, Rtgtc and ttgtc are the ground truth values.

(21) eaR=||angle(Rtc −1 Rtgtc )||22

(22) eat=||ttgtc−ttc)||22

## 5. Experimental Results and Discussion

**Figure 7:**
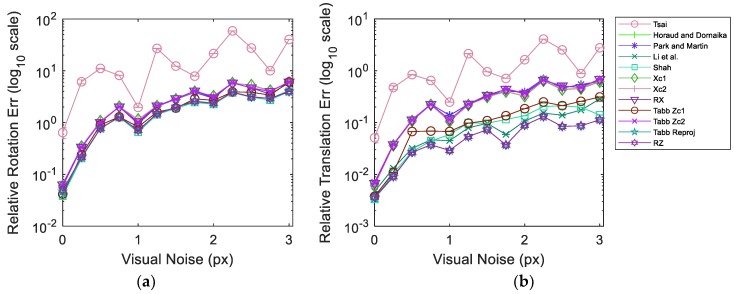

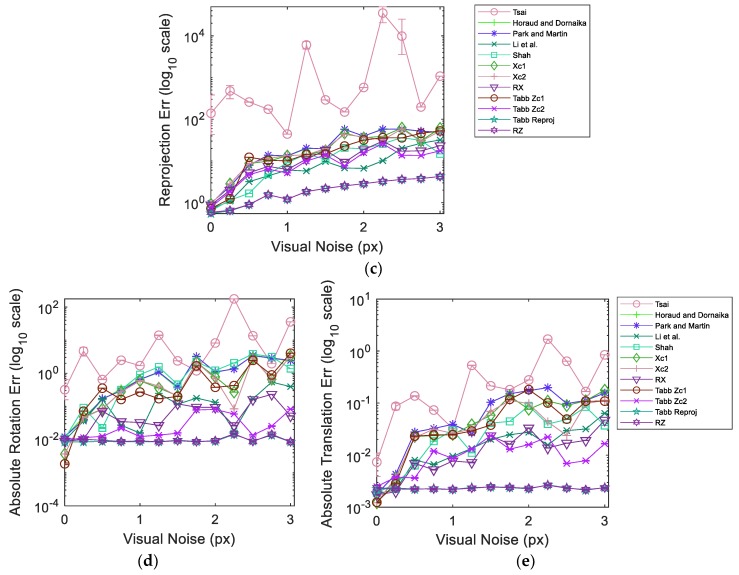
Metric error results for Dataset 5 with constant robot pose noise; (**a**) mean rotation error; (**b**) mean translation error; (**c**) reprojection error; (**d**) absolute rotation error against ground truth; (**e**) absolute translation error against ground truth.

In this section, we report the experimental results for various cases and discuss the obtained results. We tabulate the results obtained for these cases using our own and six other studies to provide a clear comparison. Table 1, Table 2, Table 3 and Table 4 summarize the results using the error metrics described in Section 4, over the datasets presented in Section 3. To elaborate on the naming, Xc1, Xc2, RX, and RZ refer to the optimization of the cost function based on Equations (5)–(7) and (13), respectively. In addition, Figure 7 illustrates the results from simulated data in dataset 5 over varying visual noise in the presence of the pseudo-realistic robotic arm pose noise. Table 2 and Table 3 shows the evaluation of the aforementioned methods on datasets 1 and 2, respectively. Both datasets vary in the use of camera lenses and robot arm poses. It can be observed that the method by Shah [8] provides a better distribution of the rotational error and hence has the lowest relative rotation error ($e_{rR}$) values, while the method by Li et al. [9] yields a comparable result. The lowest relative translation error ($e_{rt})$ varies for both datasets and is yielded by the proposed method Xc2 and Park and Martin [32]. However, for dataset 2, it seems that Xc2 has not converged properly and has obtained a local minimum. On the other hand, the method by Park and Martin [32], still yields a relatively low $e_{rt}$. Moreover, for both datasets 1 and 2, the method by Horaud and Dornaika [11] provides comparable results to Park and Martin [32].

For the reprojection root mean squared error $e_{rrmse}$, the best results are obtained using the proposed RX approach for both tests. This is aided by the fact that the recorded datasets do not have large visual errors and as a result, RX performs comparably better. Moreover, since the cost function has only one unknown transformation to minimize for, the optimizer distributes the errors more evenly for the reprojection based cost function. Other reprojection based approaches namely Tabb’s rp1 [19] and RZ achieve quite close results to RX. It is noteworthy, that in spite of being a closed-form approach, Shah [8] obtains quite good $e_{rrmse}$ that are at a competitive level to the reprojection errors based approaches.

We further study the performance of the methods using our simulated datasets. The primary difference between dataset 4 and 6 is the number and complexity of the unique camera poses for image acquisition. During experimentation, we observed that the resolution of the accuracy slightly improved with the increased number of images acquired from significantly different poses. However, none of the methods suffered significantly from comparably less information in dataset 4, therefore, we consider datasets 5 and 6 for extensive quantitative comparison of the methods. In addition to the previous tabulated results, Table 4 and Table 5 provide experimental results on simulated data with synthetic images from dataset 6. The main difference between the two tests is that the first test (Table 4) considers ideal simulated data, while the second test (Table 5) has visual and robot pose noise induced. The robot pose noise is derived from the process explained in Section 3.3, while the visual noise is selected such that the resultant reprojection error amounts to the reprojection errors of real data tests. 

Table 4 and Table 5, present two absolute errors due to the presence of ground truth information for the simulated cases. It can be observed that Tabb’s rp1 [19] achieves the least$\;e_{rR}$, $e_{rt},e_{rrmse}$ and$\;e_{at}$. Xc2 yields minimum Absolute Rotation Error ($e_{aR}$). For this dataset, the method by Park and Martin [32], failed to find a solution as it suffered from singularity. It is important to note for an ensued comparison that the proposed method RZ yields the second best results over most of the error metrics with minor differences from the least errors. This is important in a sense that all the errors are equally distributed and restricted close to their minimum values.

The backend experiments for the results in Table 5 use the same methods, metrics and dataset, as for Table 4. In agreement with the results of real data, Shah [8] yields the least $e_{rR}$ for this dataset as well. In addition to a validation on the performance of Shah [8], this indicates that a realistic amount of orientation noise is present in the system for the method to emanate similar response. The proposed method *RZ* yields the minimum $e_{rt},e_{rrmse}$ and$,\;e_{at}$ and the second best result for $e_{aR}$. Tabb Zc1 [19] obtains the minimum$\;e_{aR}$.

This comparison demonstrates that the proposed *RZ* is more robust to outliers present in the data and performs marginally better compared to Tabb’s rp1 [19] in the presence of noise.

Figure 7 shows the evaluation results for dataset 5 composed of simulated data. As before, the dataset is injected pseudo-realistic robotic arm pose noise and tested over varying realistic range of visual noise. The plots represent the averaged results over 1000 iterations in order to achieve a stable response. The 95% confidence interval from all the iterations for each experimentation point is also shown in Figure 7. It can be observed that the confidence intervals are quite narrow with the exception of the response of Tsai [3] over reprojection error metric. The narrow range of confidence interval indicates that we are 95% sure that our true mean lies somewhere within that narrow interval. Moreover, this implies that the noise introduced during different iterations is consistent in behavior and emulates a coherent response from the methods. The plot curves for each method pass through the mean values at each experimentation point. The results show that Tabb rp1 [19] and the proposed RZ are quite robust to the increments in visual noise compared to other methods over all error metrics. Moreover, at high visual noise RZ shows a slight improvement over Tabb rp1 [19]. It is noteworthy that despite the increase in relative rotation, translation and reprojection error, the absolute rotation and translation errors stay much more the same for Tabb rp1 [19] and RZ. Tsai [3] performs poorly and erratically in the presence of noise in data. In the absence of visual noise Tabb’s Zc1 [19], Xc1, RX and Shah [8] can achieve lower errors compared to Tabb rp1 [19] and RZ for multiple metrics. However, real data always contains some magnitude of visual noise due to various reasons. The presence of visual noise may affect each method differently depending on the approach used. Nonetheless, the nonlinear reprojection based methods of the formulation AX=ZB show good estimation results under visual noise and hand pose noise.

## 6. Conclusions

This study has examined the robot-world-hand–eye calibration problem in its two alternative geometrical interpretations, and has proposed a collection of novel methods. It benefits from non-linear optimizers that iteratively minimize the cost function and determine the transformations. We have conducted a comparative study to quantify the performances of optimizing over pose errors and reprojection errors. The code for the presented methods is provided as open-source for further use. Our collection of methods was evaluated with respect to state-of-the-art methods. The study also contributes new datasets for testing and validation purposes. These include subsets of three real data and three simulated data with synthetic images. Simulated data are beneficial as they provide ground truth. We have proposed a noise modeling approach to generate realistic robot TCP orientation noise to study the robustness of methods under realistic conditions. We showed that our methods perform well under different testing conditions. RX yields good results with high accuracy under realistic visual noise with respect to reprojection error. In addition, RZ is more robust to visual noise and yields more consistent results for a greater range of visual noise.

## Figures and Tables

**Table 1:** Description of the dataset acquired and generated for testing.

No.	Dataset	Data Type	Lens Focal Length [mm]	Square Size [mm]	Image Size	Robot	Poses
1	kuka_1	Real	12	20	1928 × 1208	KR16L6-2	30
2	kuka_2	Real	16	15	1920 × 1200	KR16L6-2	28
3	kuka_3	Real	12	60	1928 × 1208	KR16L6-2	29
4	CS_synthetic_1	Simulated	18	200	1920 × 1080	N/A	15
5	CS_synthetic_2	Simulated	18	200	1920 × 1080	N/A	19
6	CS_synthetic_3	Simulated	18	200	1920 × 1080	N/A	30

**Table 2:** Comparison of methods using the described error metrics for dataset 1.

Method	Evaluation Form	Relative Rotation $\left(e_{rR}\right)$	Relative Translation $\;(e_{rt})$	Reprojection Error$\;e_{rrmse}$
Tsai [3]	AXXB	0.051508	1.1855	2.5386
Horaud and Dornaika [11]	AXXB	0.051082	1.0673	2.5102
Park and Martin [32]	AXXB	0.051046	1.0669	2.5091
Li et al. [9]	AXZB	0.043268	1.6106	2.5135
Shah [8]	AXZB	**0.042594**	1.5907	2.4828
Xc1	AXXB	0.11619	7.0582	17.806
Xc2	AXXB	0.075211	**0.71369**	3.3834
Tabb Zc1 [19]	AXXB	0.051092	1.1315	2.5796
Tabb Zc2 [19]	AXZB	0.10205	3.6313	5.2324
RX	AXXB	0.076491	1.7654	**2.3673**
Tabb rp1 [19]	AXZB	0.066738	1.9455	2.4004
RZ	AXZB	0.079488	2.0806	2.4114

**Table 3:** Comparison of methods using the described error metrics for dataset 2.

Method	Evaluation Form	Relative Rotation$\;(e_{rR})$	Relative Translation$\;(e_{rt})$	Reprojection Error $\;e_{rrmse}$
Tsai [3]	AXXB	0.046162	0.48363	1.9944
Horaud and Dornaika [11]	AXXB	0.042587	0.4104	1.3804
Park and Martin [32]	AXXB	0.042639	**0.41033**	1.3807
Li et al. [9]	AXZB	0.040297	39.535	61.466
Shah [8]	AXZB	**0.04028**	0.6078	1.5767
Xc1	AXXB	1.2697	10.038	54.436
Xc2	AXXB	9.7461	24.908	197.96
Tabb Zc1 [19]	AXXB	0.61435	4.9182	16.103
Tabb Zc2 [19]	AXZB	0.48439	13.518	23.672
RX	AXXB	0.092173	0.6726	**1.1234**
Tabb rp1 [19]	AXZB	0.16515	0.84439	1.1438
RZ	AXZB	0.14824	0.81163	1.1567

**Table 4:** Comparison of methods using the described error metrics for dataset 6.

Method	Evaluation Form	Relative Rotation $\left(e_{rR}\right)$	Relative Translation $\left(e_{rt}\right)$	Reprojection Error $\;e_{rrmse}$	Absolute Rotation Error$\;(e_{aR})$	Absolute Translation Error $\;(e_{at})$
Tsai [3]	AXXB	0.65051	50.062	20.423	1.1567	8.2512
Horaud and Dornaika [11]	AXXB	0.049173	6.2428	0.60685	0.028066	2.0674
Park and Martin [32]	AXXB	NaN	NaN	NaN	NaN	NaN
Li et al. [9]	AXZB	0.031909	3.6514	0.44024	0.012108	1.0889
Shah [8]	AXZB	0.032997	1.5195	0.18418	0.021235	1.0213
Xc1	AXXB	0.051304	5.7074	0.50083	0.0079584	0.73682
Xc2	AXXB	0.051239	5.7076	0.493	**0.0075352**	0.75278
Tabb Zc1 [19]	AXXB	0.049653	5.8363	0.45621	0.01299	0.97462
Tabb Zc2 [19]	AXZB	0.033778	1.9665	0.31189	0.011335	0.69158
RX	AXXB	0.049583	5.8213	0.34127	0.01078	0.25753
Tabb rp1 [19]	AXZB	**0.031857**	**1.0829**	**0.057526**	0.0085848	**0.19154**
RZ	AXZB	0.032432	1.1072	0.05826	0.0084204	0.21121

**Table 5:** Comparison of methods using the described error metrics for dataset 6 with robot pose and visual noise.

Method	Evaluation Form	Relative Rotation$\;(e_{rR})$	Relative Translation $\left(e_{rt}\right)$	Reprojection Error $\;e_{rrmse}$	Absolute Rotation Error $\;(e_{aR})$	Absolute Translation Error$\;(e_{at})$
Tsai [3]	AXXB	34.925	2476.4	99190	28.04	747.48
Horaud and Dornaika [11]	AXXB	1.723	199.92	18.764	0.43124	47.913
Park and Martin [32]	AXXB	1.7208	199.98	18.916	0.43819	47.733
Li et al. [9]	AXZB	1.177	80.061	7.8757	0.0029485	23.272
Shah [8]	AXZB	**1.1767**	58.552	8.5123	0.51765	8.3389
Xc1	AXXB	1.7752	192.86	17.442	0.12827	37.068
Xc2	AXXB	1.8026	193.22	19.031	0.20831	40.4
Tabb Zc1 [19]	AXXB	1.7989	206.01	13.445	**0.0042828**	11.368
Tabb Zc2 [19]	AXZB	1.2571	86.844	13.891	0.050182	27.247
RX	AXXB	1.8087	204.06	12.534	0.027714	7.0139
Tabb rp1 [19]	AXZB	1.2093	44.982	1.5463	0.0075401	0.95904
RZ	AXZB	1.2079	**44.932**	**1.546**	0.0069577	**0.95845**
